# Pseudocysts of the Adrenal Gland: A Systematic Review of Existing Scientific Literature From 2000 to 2023

**DOI:** 10.7759/cureus.70528

**Published:** 2024-09-30

**Authors:** Saad Abdullah Dar, Fahad Qayyum, Arham Amir, Muhammad Ubaid Ullah Khan, Muhammad Ans Asif, Ammara Saif Ullah, Maira Jabbar Chaudhry, Hafsa Afzaal, Haseeb Mehmood Qadri

**Affiliations:** 1 General Surgery, Aziz Bhatti Shaheed Teaching Hospital, Gujrat, PAK; 2 General Surgery, Jinnah Hospital, Lahore, PAK; 3 General Surgery and Surgical Oncology, Shaikh Zayed Medical Complex, Lahore, PAK; 4 General Surgery, Ghurki Trust Teaching Hospital, Lahore, PAK; 5 General Surgery, Lahore General Hospital, Lahore, PAK; 6 Pathology, Azra Naheed Medical College, Lahore, PAK; 7 Internal Medicine, Allama Iqbal Medical College, Lahore, PAK

**Keywords:** adrenal, adrenal gland, adrenal pseudocyst, cyst, hemorrhage, pakistan, pseudocyst, pseudocyst of adrenal gland, review

## Abstract

Adrenal masses are abnormal growths in the adrenal gland, comprising entities such as pheochromocytomas, adrenal adenomas, adrenocortical carcinomas, and adrenal cysts. Pseudocysts are predominant among adrenal cysts. Due to its infrequent presentation, there are no specific guidelines present in the current literature to steer its management. In such circumstances, a systematic review of the existing literature is imperative to develop comprehensive insights and evidence-based protocols. We aimed to comprehensively analyze the clinico-radiological characteristics and management outcomes of adrenal gland pseudocysts. Human adrenal gland pseudocysts identified through imaging and histopathology, as retrieved from the PubMed search engine, were included in the study. Preferred Reporting Items for Systematic Reviews and Meta-Analyses (PRISMA) and the Joanna Briggs Institute (JBI) Critical Appraisal Checklist were used to stratify searched studies published between 2000 and 2023. A total of 39 studies were finally included, of which 36 were case reports and three case series, containing 45 patients in total. Data for clinical, radiological, histopathological, and outcome variables were collected, and descriptive analysis was carried out.

All cases presented were adults with a clear female predominance of 66.67%. About 26.67% presented with no palpable mass or clinical symptoms, while 28.89% presented with vague abdominal pain. The most prevalent computed tomography (CT) finding was a cystic lesion with calcification and/or hemorrhage and/or necrosis, occurring in 17.78% of cases. Following this, a cystic lesion with only calcification was observed in 13.33% of cases, and a well-defined cystic mass/lesion was found in 11.11% of cases. The most important indication for surgery was compression effect in 44.44%, increasing size in 20.00%, and suspicion of malignancy in another 20.00% of cases. About 64.44% underwent open surgery, while 35.55% underwent minimally invasive surgery. Most patients, 95.55% of the total, had an uneventful postoperative course without any complications. Adrenal gland pseudocyst, though rare and incidental, warrants consideration in differential diagnosis as it presents with vague symptoms and sometimes no symptoms at all. Our review of existing literature highlights the importance of surgical intervention for symptomatic or potentially malignant cysts, with en bloc adrenalectomy being the preferred approach.

## Introduction and background

The adrenal gland is a potential site for various pathological conditions, including hyperplasia, hemorrhage, and benign and malignant masses [1]. Pathological masses of the adrenals include tumors, primarily adenoma, pheochromocytoma, and adrenocortical neoplasms. According to the National Institute of Health State-of-the-Science Statement, adrenal incidentalomas are defined as clinically inapparent adrenal masses discovered inadvertently in the course of diagnostic testing or treatment for conditions not related to adrenals [2]. Adrenal cysts are rare with a reported incidence of 0.064%-0.18% [3]. Most of these lesions are either discovered incidentally or in an autopsy [3,4]. The occurrence of adrenal incidentaloma increases with age with 1% in below 30 years and increases to 3%-7% in over 70 years [4]. Besides neoplastic masses, cysts, pseudocysts, and vascular lesions do occur, though they are less common [5]. Carsote et al., in their review article involving children, described a classification for cystic lesions of the adrenal gland [6]. Accordingly, cystic lesions of the adrenals may be grouped into three main types: "pure" cystic types, parasitic cysts, and cystic part of an otherwise solid tumor usually related to a process of necrosis or hemorrhage [6]. Classical/pure type of cysts include three different subtypes. The most frequent is a vascular or endothelial cyst, which has either lymphangiomatous or hemangiomatous origin. The next prevalent type is the hemorrhagic cyst, also called pseudocyst or hemorrhagic pseudocyst [6].

Adrenal pseudocysts (APs) are cystic lesions arising within the adrenal gland surrounded by a fibrous tissue wall devoid of a recognizable lining layer [7]. Most APs are detected after they attain a large size and present with non-specific abdominal symptoms such as abdominal pain, vomiting, and compression symptoms as dominant features [8,9]. Most APs are benign, nonfunctional cystic masses that originate within the adrenal cortex or medulla. Although the pathophysiology is debatable, the pathogenesis of APs may lie in repeated episodes of trauma, infection, or bleeding [10,11]. They often present with non-specific clinical and radiological findings and hence present as a diagnostic ambiguity and are usually incidentally discovered [12,13]. A review of existing literature highlights that APs can have a variety of clinical presentations and can mimic other pathologies, which include adrenocortical cancer, pancreatic pseudocyst, hydatid/hepatic cyst, retroperitoneal cyst/cystic neoplasm, renal cyst, and pheochromocytoma [13-22]. APs can even present with features of acute abdomen and shock [23]. With a wide range of clinical presentations, there is a need to address atypical clinical findings to aid in the diagnosis of APs.

The diagnosis and management of adrenal cysts have changed largely due to the advancements in diagnostic imaging. In the recent era, because of advancements in imaging modalities, APs can be diagnosed, but imaging cannot differentiate between the benign or malignant nature of the disease [3]. Computed tomography (CT) represents the first-level imaging modality for the evaluation of adrenal lesions, since it allows a quick execution ensuring high spatial resolution [1]. Magnetic resonance imaging (MRI) can be advantageous, especially if CT scan findings are inconclusive, due to its ability to combine three-dimensional imaging with diffusion-weighted and dynamic contrast enhancement, as well as its capability for multiplane reconstruction. From the existing literature, on CT scans, APs appear as a well-demarcated, non-enhancing, hypoattenuating lesion, and on MRI, cysts are usually hyperintense on T2-weighted images [1]. However, radiological characteristics described in reported cases can be much more diverse, and there is a need to address specific radiological characteristics to diagnose APs. Clinical correlation with radiological findings is of importance as to decide between surgical excision and watchful waiting. Benign cysts or pseudocysts can be large enough to cause significant pain and discomfort. Therefore, for symptomatic relief, surgical excision or the drainage of the pseudocyst can be undertaken [3]. However, the indications to undertake a surgical approach are not well-defined.

The rationale of our systematic review is to scrutinize all reported cases of APs within the last 23 years. The published data on typical and atypical findings (clinical and radiological) and management plans followed in all studies will be analyzed. The data will then be reviewed, and a comprehensive diagnostic and management strategy for APs will be formulated.

## Review

Objective

The objective of this study is to comprehensively analyze the clinico-radiological and management outcomes of adrenal gland pseudocysts.

Methodology

This systematic review was registered on the International Prospective Register of Systematic Reviews (PROSPERO) via the identification number CRD42023495212 and conducted via Preferred Reporting Items for Systematic Reviews and Meta-Analyses (PRISMA) guidelines. A systematic exploration of PubMed using the keywords adrenal gland and/or pseudocyst was done.

Search Strategy

Boolean search operators were used as a search strategy to identify relevant literature from 2000 to 2023. The following combination of words was used: "adrenal" AND "cyst," "adrenal gland" AND "pseudocyst," "adrenal" AND "pseudo-cyst," and "adrenal" AND "pseudo cyst."

Inclusion and Exclusion Criteria

Studies, especially case reports and case series, reporting adrenal gland pseudocysts in human beings confirmed on imaging and histopathological examination were included. All animal and cadaveric studies were excluded. Editorial essays, conference abstracts, editorial reviews, and short communication were also excluded.

Search Outcome and Quality Assessment

**Figure 1:**
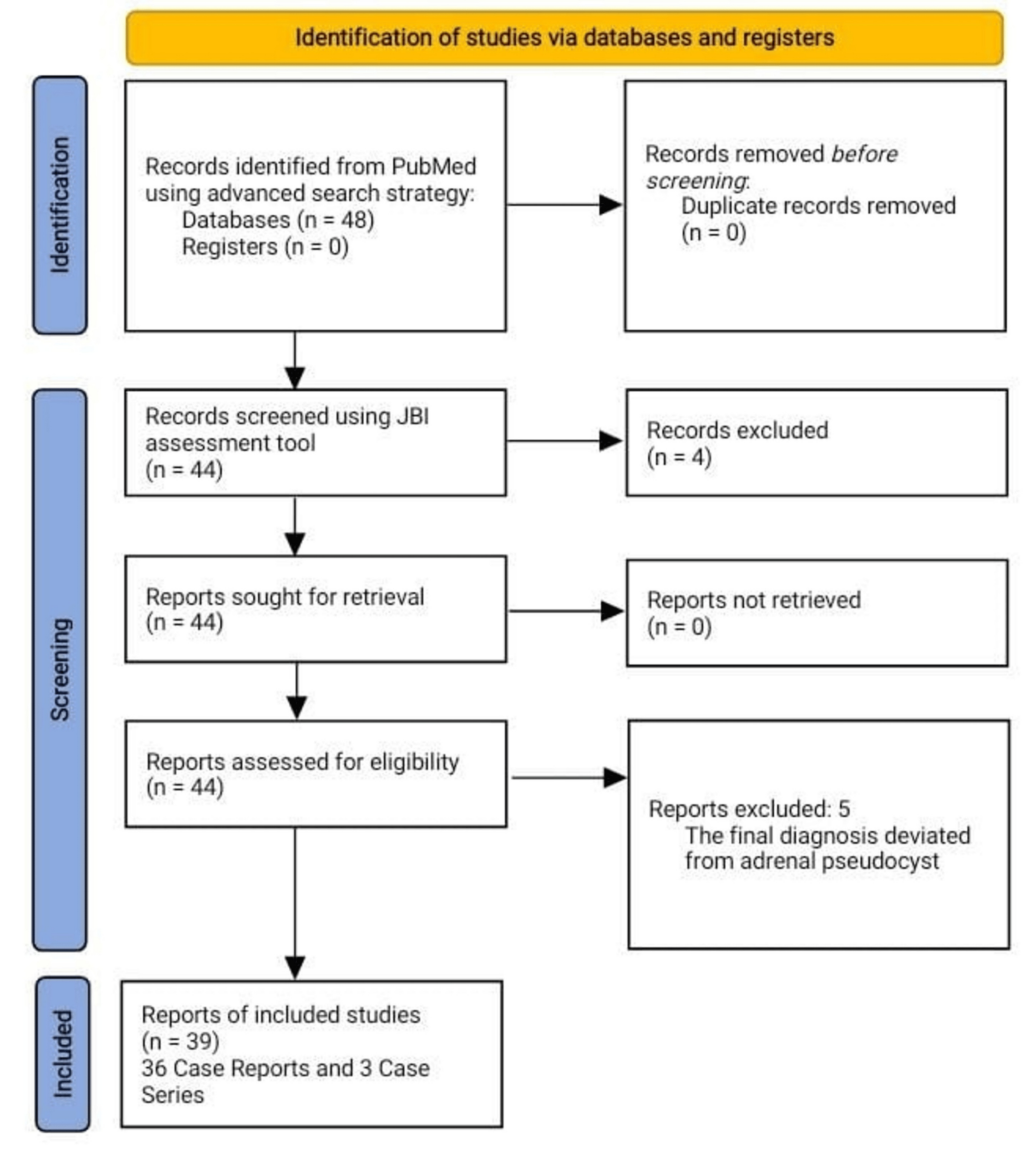
Preferred Reporting Items for Systematic Reviews and Meta-Analyses (PRISMA) flow chart for search strategy and the quality assessment of included studies. JBI: Joanna Briggs Institute

Two independent reviewers, AA and SAD, conducted the literature search and data extraction thoroughly. Discrepancies were resolved through discussion or by consulting the supervisor, HMQ. Through an extensive review of the literature, there were about 48 research articles (including case reports and case series) selected for further scrutiny and quality assessment. The quality of the included studies was evaluated using the Joanna Briggs Institute (JBI) Critical Appraisal Checklist for case series and case reports (see Appendices). The assessment criteria included study design, sample representativeness, case definition, the measurement of outcomes, and statistical analysis. Studies were rated according to predetermined criteria, with quality scores assigned accordingly. Out of 48 articles selected, 44 passed the JBI quality assessment tool, and these were forwarded for data analysis. A further five articles were excluded after data analysis as they deviated from the original diagnosis of adrenal pseudocyst. To summarize, a total of 39 study articles were analyzed (36 case reports and three case series), consisting of data on 45 patients (Figure 1).

Data Variables and Analysis

Data was extracted for patient demographics, clinical presentation, imaging findings, diagnostic methods, treatment modalities, and outcomes from included studies. Descriptive statistics, including frequencies, proportions, and means, were used to summarize the results.

Results

There was a wide geographic distribution of reported cases. The top four countries with the maximum number of cases have been mentioned, with India conducting the highest number of studies, accounting for 33.33% of the total. Japan followed with 15.30% of studies. Turkey and the United States of America both conducted four studies each, contributing 10.20% each to the overall distribution (Table 1).

**Table 1:** Summary of the articles included in a systematic review.

Author	Study Title	Year of Publication	Country of Publication
Nasir et al. [8]	Giant adrenal gland pseudo-cyst: a case report with literature review	2023	Pakistan
Parasar et al. [9]	Giant Adrenal Pseudocysts: An Enigma for Surgeons	2022	India
Olowu and Alzehairy [10]	A huge haemorrhagic suprarenal pseudocyst: an unusual presentation of a rare condition	2021	Qatar
Yokoyama et al. [11]	Differential diagnosis and laparoscopic resection of an adrenal pseudocyst: A case report	2020	Japan
Solanki et al. [12]	Cystic lesions of the adrenal gland	2023	India
Paramythiotis et al. [13]	Surgical Management of a Giant Adrenal Pseudocyst: A Case Report and Review of the Literature in the Last Decade	2018	Greece
Ates et al. [14]	A giant adrenal hemorrhagic pseudocyst mimicking a parapelvic renal cyst: A case report and review of the literature	2020	Turkey
Chue et al. [15]	Right adrenal gland pseudocyst masquerading as a large symptomatic hepatic cyst: Single incision laparoscopic (SILS) resection and a review of current literature	2018	Singapore
Bibi et al. [16]	A giant hemorrhagic adrenal pseudocyst mimicking hydatid cyst	2018	Tunisia
Isono et al. [17]	A Case of Hemorrhagic Adrenal Pseudocyst Mimicking Solid Tumor	2017	Japan
Papaziogas et al. [18]	Adrenal Pseudocyst Presenting as Acute Abdomen during Pregnancy	2006	Greece
Mahmodlou and Valizadeh [19]	Spontaneous Rupture and Hemorrhage of Adrenal Pseudocyst Presenting With Acute Abdomen and Shock	2011	Iran
Bhamidipati and Smeds [20]	Surgery for a pancreatic pseudocyst uncovers an adrenal mass instead	2010	United States of America
Kim et al. [21]	Laparoscopic resection of an adrenal pseudocyst mimicking a retroperitoneal mucinous cystic neoplasm	2009	South Korea
Bovio et al. [22]	Adrenal pseudocyst mimicking cancer: A case report	2007	Italy
Sivasankar et al. [23]	Acute Hemorrhage into Adrenal Pseudocyst Presenting with Shock: Diagnostic Dilemmas - Report of Three Cases and Review of Literature	2006	India
Ohzeki et al. [24]	Giant adrenal pseudocyst removed using robot-assisted surgery	2023	Japan
Goel et al. [25]	Cystic adrenal lesions: A report of five cases	2021	India
Moonim et al. [26]	Synchronous Microscopic Epstein-Barr Virus–Positive Diffuse Large B-Cell Lymphoma of the Adrenal and Lymphoplasmacytic Lymphoma: De Novo Disease or Transformation?	2017	United Kingdom
Geleit et al. [27]	A unique presentation of a complex haemorrhagic adrenal pseudocyst	2016	United Kingdom
Patnaik et al. [28]	All Those Liver Masses are not Necessarily from the Liver: A Case of a Giant Adrenal Pseudocyst Mimicking a Hepatic Cyst	2015	United States of America
Kodama et al. [29]	Laparoscopic Management of a Complex Adrenal Cyst	2015	Japan
Passoni et al. [30]	A Giant Adrenal Pseudocyst Mimicking an Adrenal Cancer: Case Report and Review of the Literature	2013	Switzerland
Angelico et al. [31]	Laparoscopic Adrenalectomy for Hemorrahagic Adrenal Pseudocyst Discovered During Pregnancy: Report of a Case	2013	Italy
Ujam et al. [32]	Adrenal pseudocyst: Diagnosis and laparoscopic management - A case report	2011	United Kingdom
Momiyama et al. [33]	A giant adrenal pseudocyst presenting with right hypochondralgia and fever: a case report	2011	Japan
Marwah et al. [34]	Adrenal pseudocyst mimicking cystic neoplasm of pancreatic tail	2011	India
Karaman et al. [35]	Giant hemorrhagic adrenal pseudocyst in a primiparous pregnancy: Report of a case	2011	Turkey
Stimac et al. [36]	A giant hemorrhagic adrenal pseudocyst: case report	2008	Croatia
Kar et al. [37]	Laparoscopic Resection of an Adrenal Pseudocyst	2006	United States of America
Demir et al. [38]	A Giant Adrenal Pseudocyst: Case Report and Review of the Literature	2006	Turkey
Erem et al. [39]	Large Adrenal Pseudocyst Presenting with Epigastric Distress and Abdominal Distention	2005	Turkey
Favorito et al. [40]	Traumatic rupture of adrenal pseudocyst leading to massive hemorrhage in retroperitoneum	2004	Brazil
Fan et al. [41]	Adrenal pseudocyst: A unique case with adrenal renal fusion, mimicking a cystic renal mass	2004	United States of America
Suga et al. [42]	Adrenal pseudocyst mimicking a pheochromocytoma found after a traffic accident	2003	Japan
Parshad and Kumar [43]	Pseudocyst of Adrenal Gland	2002	India
Karayiannakis et al. [44]	Giant adrenal pseudocyst presenting with gastric outlet obstruction and hypertension	2002	Greece
Basiri et al. [45]	Hypertension Secondary to an Adrenal Pseudocyst Cured by Laparoscopic Partial Adrenalectomy	2002	Iran
Ansari et al. [46]	Cost-reductive retroperitoneal excision of large adrenal pseudocyst: A case report and review of the literature	2001	India

All patients fell into the adult age group category. There were no cases identified within the pediatric age group. There was a female preponderance noted among the diagnosed cases. Out of the 45 patients, 33.33% of the cases were male, while the majority, constituting 66.67%, were female (Table 2). Among the males, the mean age was 46.46 ± 12.24 years. The mean age of females was relatively lower than that of males, 37.83 ± 14.46 years (Table 3).

**Table 2:** Gender distribution of included patients, where the total number of patients is N.

Gender	Number of Cases, n (N = 45)	Percentage Occurrence, % (n/N)
Male	15	33.33%
Female	30	66.67%

**Table 3:** Mean age and standard deviation of the included patients.

Gender	Mean Age (Years)	Standard Deviation
Male	46.46	12.24
Female	37.83	14.46

The most common symptom reported was vague abdominal pain or discomfort, which occurred in 28.89% of cases. The next common complaints were left-sided flank or abdominal pain at 24.44% and right upper quadrant (RUQ) pain at 17.78%. Notably, 11.11% of patients presented with no specific symptoms (denoted as "nil" in Table 4). Overall, the data highlights the diverse array of symptoms experienced by patients at presentation, with abdominal pain being the most prevalent (Table 4).

**Table 4:** Presenting symptoms of patients along with their percentage occurrence, where the total number of patients is N. SOB: shortness of breath

Symptoms at Presentation	Number of Cases, n (N = 45)	Percentage Occurrence, % (n/N)
Vague abdominal pain or discomfort	13	28.89%
Left-sided flank or abdominal pain	11	24.44%
Right upper quadrant pain	8	17.78%
Right flank pain	5	11.11%
Abdominal distension	4	8.89%
Nausea or vomiting	4	8.89%
Fever	3	6.67%
Upper abdominal pain	3	6.67%
Anorexia	2	4.44%
Weight loss	2	4.44%
Left testicular discomfort and swelling	1	2.22%
Severe headache	1	2.22%
Abdominal bloating	1	2.22%
Pheochromocytoma symptoms	1	2.22%
Generalized weakness	1	2.22%
Cough and SOB	1	2.22%
Early satiety	1	2.22%
Nil	5	11.11%

Out of the 45 patients, 26.67% had no palpable mass or tenderness, while 13.33% had tenderness or mass in the right upper quadrant (RUQ), and 6.67% had tenderness/mass in the left upper quadrant (LUQ) (Table 5).

**Table 5:** Presenting signs of patients along with their percentage occurrence, where the total number of patients is N. RUQ, right upper quadrant; LUQ, left upper quadrant

Signs at Presentation	Number of Cases, n (N = 45)	Percentage Occurrence, % (n/N)
No palpable mass or tenderness	12	26.67%
Tenderness or mass in the RUQ	6	13.33%
Mass/tenderness in the left lumbar region	5	11.11%
Mass involving all quadrants	4	8.89%
Pallor	4	8.89%
Abdominal tenderness only	3	6.67%
Mass or tenderness in the LUQ	3	6.67%
Mass/tenderness (undefined)	3	6.67%

The most prevalent finding on computed tomography was a cystic lesion with calcification and/or hemorrhage and/or necrosis, occurring in 17.78% of cases. A cystic lesion with only calcification was observed in 13.33% of cases, and a well-defined cystic mass/lesion was found in 11.11% of cases. A portion of the cases (13.33%) did not have any specific findings (Table 6).

**Table 6:** Findings on computed tomography (CT) scan of the abdomen and pelvis and their percentage occurrence, where the total number of patients is N.

Common Findings on CT	Number of Cases, n (N = 45)	Percentage Occurrence, % (n/N)
Cystic lesion with calcification and/or hemorrhage and/or necrosis	8	17.78%
Cystic lesion with calcification	6	13.33%
Well-defined cystic mass/lesion	5	11.11%
Hyperdense cystic mass	3	6.67%
Hypodense lesion	3	6.67%
Cystic lesion with smooth/regular margins	2	4.44%
Cystic lesion with enhancement	2	4.44%
Hypodense cystic mass with hyperdense areas in mass	2	4.44%
Cystic lesion with calcification and enhancement	1	2.22%
Cystic mass with minimal calcification and septation	1	2.22%
Cystic lesion with multiple septations	1	2.22%
Solid lesion with less than 25% cystic component showing enhancement	1	2.22%
Solid lesion with less than 25% solid component without enhancement	1	2.22%
Hypodense cystic mass with hyperdense areas in mass	2	4.44%
Mass with scattered calcification	1	2.22%
Giant cystic mass	1	2.22%
Thin-walled cystic mass containing non-enhancing fluid	1	2.22%
Not given	6	13.33%

Among the documented magnetic resonance imaging (MRI) findings, the most common features were hyperintensity on T1 and T2 images accounting for 11.11% of cases, 6.67% showed low intensity on T1 and high intensity on T2, 4.44% showed hyperintensity on T1, and 4.44% showed hyperintensity on T2. The majority of cases (71.11%) had undocumented MRI findings (Table 7).

**Table 7:** MRI findings in patients and their percentage occurrence, where the total number of patients is N. MRI: magnetic resonance imaging

Common Findings on MRI	Number of Cases, n (N = 45)	Percentage Occurrence, % (n/N)
Hyperintensity on T1 and T2	5	11.11%
Low intensity on T1 and high on T2	3	6.67%
Hyperintensity on T1	2	4.44%
Hyperintensity on T2	2	4.44%
Low intensity on T1 and high intensity on T2 and diffusion-weighted images	1	2.22%
Not given	32	71.11%

The most prevalent reasons to resort to surgery were symptomatic or compressive masses in 44.44% of cases and the suspicion of malignancy in 20.00% of cases. Other indications included an increase in size larger than 5 cm in 20.00%, shock in 8.89%, and the rupture of a cyst or collection in the retroperitoneal area in 6.67% of cases (Table 8). The data indicates that 57.78% of the lesions were on the left side, while 42.22% were on the right side (Table 9). About 64.44% underwent an open surgical approach, while 35.55% underwent a minimally invasive approach (Table 10).

**Table 8:** Indication of surgery in included patients and their percentage occurrence, where the total number of patients is N.

Indication of Surgery	Number of Cases, n (N = 45)	Percentage Occurrence, % (n/N)
Compressive or symptomatic mass	20	44.44%
Increased size of >5 cm	9	20.00%
Malignancy suspicion	9	20.00%
Shock	4	8.89%
Cyst rupture or retroperitoneal collection	3	6.67%

**Table 9:** Lesion distribution by side and their percentage occurrence, where the total number of patients is N.

Side of Lesion	Number of Cases, n (N = 45)	Percentage Occurrence, % (n/N)
Left-sided	26	57.78%
Right-sided	19	42.22%

**Table 10:** Type of surgery performed in patients and their percentage occurrence, where the total number of patients is N.

Type of Surgery	Number of Cases, n (N = 45)	Percentage Occurrence, % (n/N)
Open	29	64.44%
Minimally invasive	16	35.55%

Thirteen studies have documented cyst volume for 13 patients. The mean volume was 1906.36 ± 1814.48 cm^3^. Eighteen studies documented cyst areas. The mean area was 140.78 ± 97.47 cm^2^. Twelve studies reported a single dimension of cyst. The mean size was 12.53 ± 9.15 cm. Out of 45 patients, 2.22% had lesions involving the adrenal cortex, 2.22% had lesions involving the adrenal medulla, and 2.22% had lesions involving both the adrenal cortex and medulla. In 42 patients (93.33%), no site of lesion was mentioned. The specimens that were removed showed a variety of gross findings. The most common of these was a cyst filled with hemorrhagic contents, which appeared in 35.5% of cases. Unilocular cysts were the second most common finding, appearing in 11.11% of cases, followed by multi-lobulated cysts filled with straw-colored fluid, which occurred in 6.67% of cases (Table 11).

**Table 11:** Gross findings of the excised specimen and their percentage occurrence, where the total number of patients is N.

Gross Findings	Number of Cases, n (N = 45)	Percentage Occurrence, % (n/N)
Cyst containing hemorrhagic contents	16	35.5%
Unilocular cyst	5	11.11%
Multi-lobulated cyst with straw-colored fluid	3	6.67%
Cyst containing light-brown serous fluid	2	4.44%
Cyst with yellowish necrotic material	2	4.44%
Well-encapsulated cyst	2	4.44%
Thick-walled cyst with yellow-brown material	2	4.44%
Thick-walled cyst with viscous brown fluid	1	2.22%
Mass with dark-red appearance	1	2.22%
Unilocular cyst with rough internal surface	1	2.22%
Cyst with gray-brown membranous tissue	1	2.22%
Multicystic adrenal mass	1	2.22%
Ruptured cyst	1	2.22%
Thin-walled adrenal cyst	1	2.22%

The diagnosis of pseudocyst was confirmed on histopathology. There were certain specific histological findings. The most common features were pseudocyst with hemorrhagic content, a fibrous wall with or without calcification, and combined features of calcification, a fibrous wall with inflammatory cells, cholesterol clefts, and hemosiderophages accounting for 22.22%, 22.22%, and 13.33%, respectively; 6.67% had foamy macrophages, while 4.44% had cyst without epithelial coverage (Table 12).

**Table 12:** Histopathological findings of the excised specimen and their percentage occurrence, where the total number of patients is N.

Histopathological Findings	Number of Cases, n (N = 45)	Percentage Occurrence, % (n/N)
Pseudocyst with hemorrhagic content	10	22.22%
Pseudocyst with fibrous wall with/without calcification	10	22.22%
Pseudocyst with fibrous wall and calcification plus inflammatory cells, cholesterol clefts, and hemosiderophages	6	13.33%
Pseudocyst with fibrous wall and pigmented foamy macrophages	3	6.67%
Cyst without epithelial coverage	2	4.44%
Pseudocyst with hemorrhage and calcification	1	2.22%
Pseudocyst with hemorrhage and surrounded by clear adrenocortical cells	1	2.22%
Pseudocyst with hematic content and granulated histiocytes	1	2.22%
Pseudocyst with fibrous wall plus an aggregate of small lymphocytes and plasma cells	1	2.22%
Not given	10	22.22%

Only two individuals (4.44% of the total) experienced complications. One patient (2.22%) developed a fever after the surgical procedure, while another individual (2.22%) had paroxysmal atrial fibrillation. On the other hand, most patients (95.55% of the total), comprising 43 cases, had an uneventful postoperative course without any complications. Most of the patients, accounting for 37.78% of the total, stayed in the hospital for a period ranging from one to six days (Table 13 and Table 14).

**Table 13:** Postoperative complications in patients and their relevant percentage occurrence, where the total number of patients is N.

Postoperative Complications	Number of Cases, n (N = 45)	Percentage Occurrence, % (n/N)
Fever	1	2.22%
Paroxysmal atrial fibrillation	1	2.22%
Uneventful	43	95.55%

**Table 14:** Duration of hospital stay of patients and their percentage occurrence, where the total number of patients is N.

Length of Hospital Stay	Number of Cases, n (N = 45)	Percentage Occurrence, % (n/N)
1-6 days	17	37.78%
7-12 days	9	20.00%
Not given	19	42.22%

Out of the total cases, 46.67% experienced hemorrhage into the cyst cavity, while an equal percentage of cases (46.67%) did not exhibit this complication. There was no information on the remaining 6.67% (Table 15).

**Table 15:** Hemorrhage into cyst cavity and their percentage occurrence, where the total number of patients is N.

Hemorrhage Into Cyst Cavity	Number of Cases, n (N = 45)	Percentage Occurrence, % (n/N)
Yes	21	46.67%
No	21	46.67%
Not given	3	6.67%

Discussion

Retroperitoneal cysts are rare lesions of the retroperitoneum that can be classified into neoplastic and non-neoplastic based on their malignant potential. The neoplastic cysts include serous/mucinous cystadenoma, Mullerian cyst, cystic teratoma, cystic lymphangioma, cystic mesothelioma, tailgut cyst, omental/mesenteric cyst, and epidermoid cyst. Among non-neoplastic cysts are urinoma, hematoma, pancreatic pseudocysts, non-pancreatic pseudocysts, and lymphocele [47]. Adrenal gland cysts are the rare nonfunctioning asymptomatic cystic lesions arising within the adrenal gland [8,14]. The incidence of adrenal cysts as demonstrated by various autopsy series is around 0.064%-0.18% and 5%-6% in clinical series [8,12]. Around 7% of adrenal cysts have a malignant potential, and therefore, careful assessment is required preoperatively to rule out malignancy [24].

Cystic adrenal lesions can be broadly classified as pseudocysts, endothelial cysts, epithelial cysts, and parasitic cysts with endothelial cysts being the most common and parasitic cysts being the least common [29]. Endothelial cysts can be further subdivided into angiomatous, lymphangiomatous, and hamartomatous cysts [48]. Pseudocyst is the most common type that is discovered intraoperatively and accounts for 32%-80% of all adrenal cysts [8,32]. The etiology of adrenal pseudocysts is not clearly known; however, they may result from hemorrhage into the adrenal gland with the subsequent cystic degeneration of the hematoma [30] and the cystic degeneration of adrenal tissue or already existing tumorous mass [24]. Hemorrhage into adrenal tissue can result from trauma, anticoagulant therapy, complicated pregnancy, sepsis, or trauma, with trauma being the most common cause resulting in unilateral hemorrhage into the adrenal gland [1].

Adrenal pseudocysts are found more abundantly in females as compared to males as shown in a study conducted by Zheng et al. in which there were 14 females and nine males [5]. This is similar to our study, which enlists 30 females and 15 males. However, Chien et al. conducted a study on adrenal cystic lesions and found more males (14) as compared to females (11) [49]. In contrast to adrenal cysts, renal cysts are found more commonly in males as compared to females as shown in a study conducted in Pomerania where the prevalence of renal cysts was found to be higher in males (34%) as compared to females (21%) [50]. Though the adrenal glands and kidneys lie in apposition to each other, the variety of cystic lesions, their gender and age predilection, and clinical manifestations are still not well-understood. The mean age in our study was 42.14 years. As already described in the literature, these lesions are common in the third to sixth decade of life, and one possible explanation for this is that with advancing age, atherosclerosis increases, and local tissue ischemia may be responsible for the development of such masses [4]. Zheng et al. conducted a study on adrenal cystic lesions and found a mean age of 49 years for 23 cases of pseudocyst out of a total of 55 cases of adrenal cysts, which is comparable to our study [5]. Lymphangiomatous cysts, in contrast, affect younger adults, and a mean age of 40 was found in the same study by Zheng et al. [5]. 

In a study by Neri and Nance in 1999, it was found that 34% of all adrenal cysts are found by chance when patients undergo imaging studies for other reasons and 39% of the cysts present with abdominal pain and/or mass [51]. In 12 patients of our study, the pseudocyst was identified incidentally. They had no signs or symptoms whatsoever. In a study by Gubbiotti et al., all 18 cases of adrenal cysts were found to be incidental [3]. The major presenting sign was tenderness and abdominal mass, while the most common presenting symptom was vague abdominal/left flank pain or discomfort like in a study by Zheng et al. in which vague abdominal/flank/back pain was found to be the most common presenting complaint [5]. Similarly in a study by Bellantone et al., out of 12 patients, six presented with abdominal pain, one with a palpable mass, and one with hemorrhagic shock, making abdominal pain and mass the most common presenting features [52]. Cystic adrenal lesions may cause pheochromocytoma-like symptoms due to catecholamine elevation, which probably occurs due to cysts pushing the adrenal medulla and causing transient elevation [53]. However, these symptoms usually resolve after the surgical removal of cysts. In our review, one out of 45 patients had pheochromocytoma-like symptoms, which resolved after surgery.

These lesions are mostly unilateral; however, in 8%-15% of cases, they can be bilateral [54]. In our study, all cases were unilateral with most of the lesions being on the left side as compared to the right side. This is similar to the findings by Gubbiotti et al. who conducted a study on adrenal cystic lesions and found 10 cases of cysts on the left side versus six cases of cysts on the right side [3]. However, Zheng et al. conducted a study that contrasted our results with 13 cases on the right side and 10 cases on the left side [5].

On CT scan, the most common findings were cystic mass with calcification, hemorrhage, and necrosis similar to the results of a study by Chien et al., which found that pseudocysts appeared as suprarenal masses having mixed density, central necrosis or cystic change, and heterogeneous enhancement on CT scan [49]. The calcification of cysts occurs due to the reason that when tissue damage and inflammation occur, calcium gets released from intracellular stores such as the mitochondria and endoplasmic reticulum. This combined with membrane damage allows the extracellular calcium to rush into cells, resulting in the deposition of calcium inside cells [55]. The cysts of retroperitoneal organs are comparable in terms of their presentation. Like adrenal cysts, simple renal cysts appear as hypodense non-enhancing lesions on CT, while complex renal cysts may need contrast administration for characterization. Pancreatic pseudocyst appears to have fluid density with a smooth well-defined wall that may be too thin to be undetectable or thick enough to display contrast enhancement [56,57]. In our review of adrenal pseudocysts, the major finding on MRI was the presence of hyperintensity on T1- and T2-weighted images or low intensity on T1 images and high intensity on T2 images, which is comparable to the pancreatic pseudocysts that generally exhibit T1 hypointensity and T2 hyperintensity. Hemorrhage or the accumulation of proteinaceous fluid may promote T1 hyperintensity in the case of pancreatic pseudocysts [56]. Similarly, renal cysts appear as hyperintense on T2-weighted images and hypointense on T1-weighted images without abnormal contrast enhancement [57].

In our review, out of 45 patients, one patient had a lesion involving the adrenal cortex, one patient had a lesion involving the adrenal medulla, and one patient had a lesion involving both. For the rest of the patients, no location was documented. Koperski et al. did a study in 2018, and out of a total of 37 patients, 35 patients had cysts involving the adrenal gland, and two patients had extra-adrenal cystic locations [53].

Small asymptomatic adrenal cystic lesions can be managed conservatively with follow-up through contrast-enhanced computed tomography (CECT) scan or magnetic resonance imaging (MRI) scan [12]. Surgical intervention is required if the cyst is symptomatic, functional, and greater than 5 cm or has a solid component suggestive of malignant potential [12]. Uncomplicated cysts can be managed conservatively with aspiration instead of excision [48]. En bloc adrenalectomy is the procedure of choice [29].

Just like adrenal cysts, asymptomatic pancreatic cysts can be managed conservatively. Intervention is required for complicated pseudocysts such as those compressing on surrounding structures such as the bile duct, causing gastric or duodenal obstruction; pseudocysts associated with pancreatic ascites or pancreatic pleural fistula; infected pseudocyst of the pancreas; hemorrhage into pseudocyst; symptomatic pseudocysts presenting with vomiting, early satiety, abdominal pain, etc.; pseudocysts greater than 6 cm in size; and extrahepatic pseudocysts [56]. Likewise, for renal cysts, Bosniak class 1 and 2 cysts need no intervention as they are benign. Bosniak class 2F needs follow-up due to some risk of malignancy, while class 3 cysts need surgical excision due to high association with malignancy [57].

In the majority of the cases in our review, surgery was performed due to the cystic mass being large enough to compress the surrounding structures and cause symptoms, while in 20% of cases, it was due to the suspicion of malignancy. Adrenalectomy en bloc, through a laparoscopic approach, is preferred for all symptomatic functional cases; however, an open surgical approach can be used if there is a suspicion of malignancy to avoid seeding the surrounding tissues [52]. In our study, 29 patients underwent open surgical removal, while the remaining 16 underwent adrenalectomy through a minimally invasive approach.

In 16 cases of our review, the cyst contained hemorrhagic contents. The pseudocysts appeared to be encapsulated, thick-walled, and containing fluids of varying varieties mostly yellowish or brownish indicating old hemorrhage. This is similar to the findings by Gubbiotti et al. who found the pseudocysts to contain hemorrhagic contents, while in the other 10 cases, fibrous tissue was found in the cyst wall with or without calcification. In addition, there were inflammatory cells, cholesterol clefts, and pigmented foamy macrophages on histopathological examination. These findings are consistent with the results of our systematic review [3]. In comparison to the adrenal pseudocysts, lymphangiomatous cysts, which are a type of endothelial cysts, are characterized by small lymphatic channels lined with flat, non-proliferative endothelial cells resembling capillaries with walls containing lymphoid aggregates as found in a study by Zheng et al. These cysts contain proteinaceous fluid devoid of significant red blood cells [5]. Likewise, angiomatous cysts, which are also a subtype of endothelial cysts, presented thin-walled, cystic spaces characterized by a solitary layer of unremarkable, flattened endothelial cells with one case exhibiting localized papillary endothelial hyperplasia [5].

Most of the 43 patients had an uneventful recovery with one patient developing a fever and the other person developing paroxysmal atrial fibrillation. In a study by Bellantone et al., one out of 12 patients developed acute cholecystitis in the postoperative period [52]. Akkuş et al. studied 229 patients with adrenal cysts, 84 patients had adrenalectomies, and only two patients developed postoperative complications. One patient developed a pancreatic fistula, and another person developed a surgical site infection [4].

Most of the patients stayed in the hospital for 1-6 days postoperatively. The mean postoperative hospital stay was 5.53 days comparable to results by Bellantone et al. who found the mean postoperative hospital stay to be nine days [52]. None of the patients in our review had disease recurrence after adrenalectomy. One out of 22 patients with adrenal cysts showed local recurrence at 12 months after partial adrenalectomy for cystic adrenal lesions, found in a study by Erickson et al. [58]. Recurrence after the excision of the retroperitoneal cyst may occur if surgical excision is incomplete. The true incidence of recurrence is not known exactly; however, one series roughly estimates it around 25% following the complete excision of pancreatic cystic neoplasm without invasion. Lee [59] and Maurya et al. [60] also documented no recurrence in the five-year interval in their study regarding pancreatic and retroperitoneal cysts, respectively. This correlation suggests that despite the adrenal pseudocyst being a relatively separate entity, the behavior of the cystic lesions is similar and surgical resection is effective only when complete pseudocyst is excised. 

This review provides a comprehensive overview of cystic adrenal lesions and their management compiling a significant amount of data from various studies. It provides detailed insights into various aspects of cystic adrenal lesions, including classification, etiology, presentation, imaging features, management strategies, and their outcomes while simultaneously comparing our findings with those of previous studies to get a broader perspective about these lesions. As with any systematic review, there is a risk of bias in the selection and interpretation of studies included in this study, and the included studies may have different study designs, different patient populations, and different diagnostic criteria, which may influence the generalizability of the findings of this study. There may be a publication bias due to the published studies with positive findings being included in this review.

Clinical Recommendations

**Figure 2:**
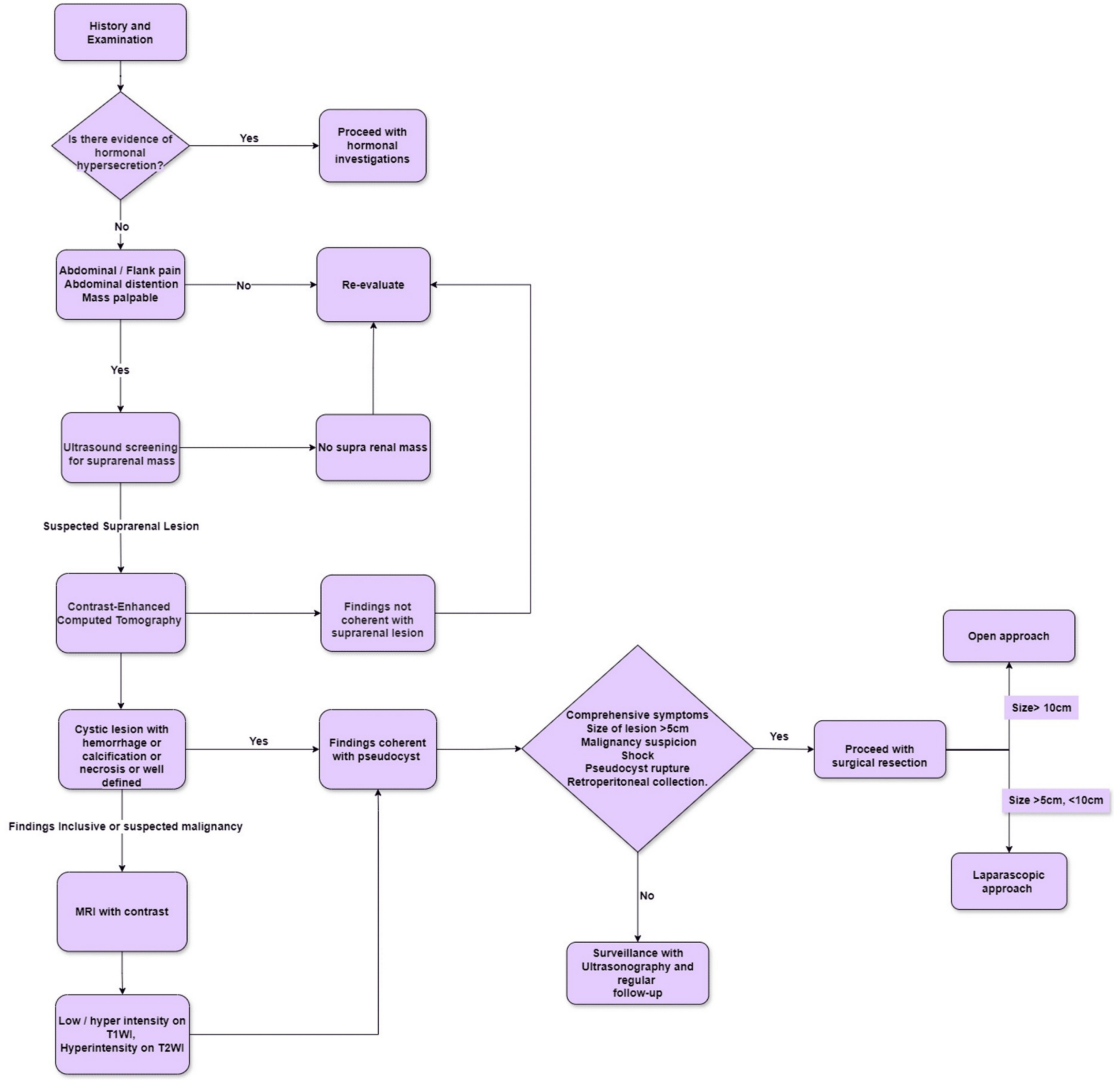
Managerial algorithm of pseudocysts of adrenal gland proposed by authors. MRI, magnetic resonance imaging; T1WI, T1-weighted image; T2WI, T2-weighted image

Using the results of the review of 39 articles, we can adequately come to a proposed plan for the diagnosis and management of APs. This is illustrated in the flowchart below (Figure 2). Certain clinical recommendations can be placed forward in concordance with our review. Long-standing vague abdominal symptoms should be correlated with detailed history and surveillance radiography. APs should undergo surgical resection if they present with features of shock, rupture, or hemorrhage in an emergency setting. In the elective setting, if the size is greater than 5 cm, compressive symptoms are present, or if there is suspected intralesional malignancy, a thorough radiographic investigation should be performed, and definitive complete surgical excision of the pseudocyst should be planned. A complete resection should be done to prevent recurrence. A minimally invasive approach should be used for symptomatic lesions of sizes between 5 cm and 10 cm, while an open surgical approach should be used for lesions greater than 10 cm or with suspected malignancy.

## Conclusions

Adrenal gland cysts are rare entities that are often discovered incidentally during imaging studies. While they are mostly asymptomatic, they can manifest with vague abdominal symptoms or complications such as hemorrhage or infection. With the increasing use of imaging modalities such as CT or MRI, these lesions are being detected more often. Management strategies vary depending on the size, symptoms, and malignant potential of the cysts. It may include conservative management, the aspiration of a cyst, or partial or total adrenalectomy. Our review of existing literature highlights the importance of surgical intervention for symptomatic or potentially malignant cysts, with en bloc adrenalectomy being the preferred approach. Further research is required to explore the underlying mechanisms of cyst formation and to refine diagnostic and therapeutic strategies.

## Appendices

Table 16 and Table 17 show the Joanna Briggs Institute Critical Appraisal Checklist for the included case reports and case series.

**Table 16:** Joanna Briggs Institute Critical Appraisal Checklist for the included case reports. Q1: Were the patient's demographic characteristics clearly described? Q2. Was the patient's history clearly described and presented as a timeline? Q3. Was the current clinical condition of the patient on presentation clearly described? Q4. Were diagnostic tests or assessment methods and the results clearly described? Q5. Was the intervention(s) or treatment procedure(s) clearly described? Q6. Was the post-intervention clinical condition clearly described? Q7. Were adverse events (harms) or unanticipated events identified and described? Q8. Does the case report provide takeaway lessons? Y, yes; N, no; U, unclear

Author	Q1	Q2	Q3	Q4	Q5	Q6	Q7	Q8	Overall Appraisal
Nasir et al. [8]	Y	Y	U	Y	Y	Y	Y	Y	Included
Olowu and Alzehairy [10]	Y	U	Y	Y	Y	Y	U	U	Included
Yokoyama et al. [11]	Y	N	U	Y	Y	Y	U	Y	Included
Solanki et al. [12]	Y	Y	U	U	Y	Y	U	Y	Included
Paramythiotis et al. [13]	Y	U	Y	Y	Y	Y	N	N	Included
Ates et al. [14]	Y	Y	U	Y	Y	Y	N	U	Included
Chue et al. [15]	Y	Y	Y	Y	Y	Y	Y	Y	Included
Bibi et al. [16]	Y	U	U	Y	Y	Y	Y	Y	Included
Isono et al. [17]	Y	Y	U	Y	Y	Y	Y	Y	Included
Papaziogas et al. [18]	Y	Y	Y	Y	Y	Y	Y	Y	Included
Mahmodlou and Valizadeh [19]	Y	U	Y	Y	Y	Y	Y	Y	Included
Bhamidipati and Smeds [20]	Y	Y	Y	Y	Y	Y	Y	Y	Included
Kim et al. [21]	Y	Y	Y	Y	Y	Y	Y	Y	Included
Bovio et al. [22]	Y	Y	Y	Y	Y	Y	Y	Y	Included
Ohzeki et al. [24]	Y	Y	Y	Y	Y	Y	U	N	Included
Moonim et al. [26]	Y	Y	U	Y	Y	Y	Y	Y	Included
Geleit et al. [27]	Y	Y	Y	Y	Y	Y	Y	Y	Included
Patnaik et al. [28]	Y	Y	Y	Y	Y	N	N	Y	Included
Kodama et al. [29]	Y	N	N	Y	Y	Y	Y	Y	Included
Passoni et al. [30]	Y	Y	Y	Y	Y	Y	Y	Y	Included
Angelico et al. [31]	Y	Y	Y	Y	Y	Y	Y	Y	Included
Ujam et al. [32]	Y	Y	Y	Y	Y	Y	Y	Y	Included
Momiyama et al. [33]	Y	U	Y	Y	Y	Y	Y	Y	Included
Marwah et al. [34]	Y	Y	Y	Y	Y	Y	Y	Y	Included
Karaman et al. [35]	Y	Y	Y	Y	Y	Y	Y	Y	Included
Stimac et al. [36]	Y	Y	Y	Y	Y	Y	Y	Y	Included
Kar et al. [37]	Y	Y	Y	Y	Y	Y	Y	Y	Included
Demir et al. [38]	Y	Y	Y	Y	Y	N	N	Y	Included
Erem et al. [39]	Y	U	Y	Y	Y	Y	Y	U	Included
Favorito et al. [40]	Y	Y	Y	Y	Y	N	N	Y	Included
Fan et al. [41]	Y	Y	Y	Y	Y	N	N	Y	Included
Suga et al. [42]	Y	Y	Y	Y	Y	U	U	Y	Included
Parshad and Kumar [43]	Y	Y	Y	Y	Y	Y	Y	Y	Included
Karayiannakis et al. [44]	Y	Y	Y	Y	Y	Y	Y	Y	Included
Ansari et al. [46]	Y	Y	U	Y	Y	N	N	Y	Included

**Table 17:** Joanna Briggs Institute Critical Appraisal Checklist for included case series. Q1. Were there clear criteria for inclusion in the case series? Q2. Was the condition measured in a standard, reliable way for all participants included in the case series? Q3. Were valid methods used for the identification of the condition for all participants included in the case series? Q4. Did the case series have a consecutive inclusion of the participants? Q5. Did the case series have a complete inclusion of the participants? Q6. Was there clear reporting of the demographics of the participants in the study? Q7. Was there clear reporting of the clinical information of the participants? Q8. Were the outcomes or follow-up results of cases clearly reported? Q9. Was there clear reporting of the presenting site(s)/clinic(s) demographic information? Q10. Was statistical analysis appropriate? Y, yes; U, unclear

Author	Q1	Q2	Q3	Q4	Q5	Q6	Q7	Q8	Q9	Q10	Overall Appraisal
Parasar et al. [9]	Y	Y	Y	Y	Y	Y	Y	Y	Y	Y	Included
Sivasankar et al. [23]	Y	Y	Y	U	Y	Y	Y	Y	Y	Y	Included
Goel et al. [25]	Y	Y	Y	Y	Y	Y	Y	Y	Y	Y	Included
